# The finishing touches: the role of friction and roughness in haptic perception of surface coatings

**DOI:** 10.1007/s00221-020-05831-w

**Published:** 2020-05-23

**Authors:** Lisa Skedung, Kathryn L. Harris, Elizabeth S. Collier, Mark W. Rutland

**Affiliations:** 1 RISE Research Institutes of Sweden, Malvinas väg 3, 114 28 Stockholm, Sweden; 2 Department of Chemistry, KTH Royal Institute of Technology, Drottning Kristinas väg 51, 114 28 Stockholm, Sweden

**Keywords:** Touch, Haptic perception, Roughness, Psychotribology, Wood furniture surfaces, Friction

## Abstract

Humans are extraordinarily skilled in the tactile evaluation of, and differentiation between, surfaces. The chemical and mechanical properties of these surfaces are translated into tactile signals during haptic exploration by mechanoreceptors in our skin, which are specialized to respond to different types of temporal and mechanical stimulation. Describing the effects of measurable physical characteristics on the human response to tactile exploration of surfaces is of great interest to manufacturers of household materials so that the haptic experience can be considered during design, product development and quality control. In this study, methods from psychophysics and materials science are combined to advance current understanding of which physical properties affect tactile perception of a range of furniture surfaces, i.e., foils and coatings, thus creating a tactile map of the furniture product landscape. Participants’ responses in a similarity scaling task were analyzed using INDSCAL from which three haptic dimensions were identified. Results show that specific roughness parameters, tactile friction and vibrational information, as characterized by a stylus profilometer, a Forceboard, and a biomimetic synthetic finger, are important for tactile differentiation and preferences of these surface treatments. The obtained dimensions are described as distinct combinations of the surface properties characterized, rather than as ‘roughness’ or ‘friction’ independently. Preferences by touch were related to the roughness, friction and thermal properties of the surfaces. The results both complement and advance current understanding of how roughness and friction relate to tactile perception of surfaces.

## Introduction

Capable of differentiating between surfaces with topographical differences of only nanometers, humans are exquisitely sensitive to tactile information (Skedung et al. 2013). Tactile perception begins with some interaction between the skin and external stimuli to which mechanoreceptors in the skin respond. Although often caused by contact with a solid surface, humans are also sensitive to a cool breeze on a summer day, kinesthetic cues arising from movement through air (Collier and Lawson 2016) or a drop of hot water erroneously encountering the skin while making a cup of coffee. Even bending of the hairs on hairy skin can give rise to a perceptual experience (Gibson 1962). One of the most sensitive areas on the human body is the fingertips (Lederman and Klatzky 2009), where mechanoreceptors for detecting short and high wavelength vibrations as well as elastic deformations to the skin are numerous and densely packed. These mechanoreceptors are specialized for their specific perceptual function (Johnson 2001) and are coupled to different nerve afferents with differing temporal response profiles (Fleming and Luo 2013).

Humans have evolved to experience many different mechanical and temporal aspects of touch, and are able to describe haptic perception of surfaces in complex ways, often using the concept of dimensional space as a metaphor for differences between these sensations (for a review of how many and which tactile dimensions may be most relevant for human touch, see Okamoto et al. (2013). Unlike mathematical descriptions of space, however, haptic dimensions are fluid and not necessarily orthogonal (Guest et al. 2011). This is immediately demonstrable by examining the concepts of ‘rough and smooth’, ‘sticky and slippery’, and ‘hard and soft’. Even casual contemplation suggests that there is plenty of overlap and dependence of these descriptors on each other, e.g., is a smooth surface sticky, or slippery? Are these two concepts really opposites? Is a slippery surface necessarily smooth? Is softness the opposite of hardness? Of roughness? Of course, the answer is always that *it depends*.

Nonetheless, roughness/smoothness (Ballesteros et al. 2005; Guest et al. 2011; Skedung et al. 2011, 2013, 2018; Yoshida 1968) and stickiness/slipperiness (Guest et al. 2011; Hollins et al. 1993, 2000; Skedung et al. 2013, 2018) are often assigned as the primary dimensions of perception. There is still, however, a general lack of work describing the interactions between these dimensions, and the nuances of their description using physical parameters. Surface roughness is more than a linear scale, and more than just height parameters are required to accurately characterize a texture. Humans are capable of detecting extremely small variations in surface texture (Skedung et al. 2013), differences in surface chemistry in the absence of roughness differences (Carpenter et al. 2018; Kuroki et al. 2017; Skedung et al. 2018) and the vibratory signals generated upon interaction, which are known to contribute especially to the perception of surfaces with fine textures (Bensmaia and Hollins 2003; Ding et al. 2017; Hollins et al. 2000). This would seem to imply that extensive characterization of surface texture, the friction and vibrational aspects of the finger-surface interaction and, crucially, how these relate to each other, are required to expand what is known about haptic experiences.

This particularity of tactile sensation translates into distinctions and preferences that are of great interest to companies who wish to fine-tune their products, giving consideration to the tactile experience delivered to the customer (Spence 2019; Spence and Gallace 2011). Thus, identifying parameters which are measurable in a laboratory setting that relate to human responses is valuable, because this can provide an understanding of how the parameters that are controllable during the production process can influence the tactile responses to their product. This is particularly relevant when this understanding might complement, or even replace, costly participant panel studies.

In this study, a broad suite of existing furniture finishes using standard quantification were perceptually evaluated, where participants were asked to assess the haptic similarity of each possible pair of stimuli while blindfolded. They also provided preference rankings, first by touch alone (while blindfolded) and then while being permitted to see the samples while feeling them. The data from the participant study was then analysed in conjunction with the physical characterization data to determine which parameters might describe perceived differences between surfaces that vary in texture and composition. Roughness and tactile friction features were characterized with a stylus profilometer and a Forceboard™. In addition, the samples were also characterized using a SynTouch Biotac Toccare™, a biomimetic system which duplicates human exploratory movements and, using multimodal sensors, characterizes the sliding contact (Xu and Fishel 2013). This instrument evaluates surfaces using 15 descriptors, or dimensions, pertaining to perceived roughness (macro and micro texture), friction (static and kinetic), adhesion, stiffness (compliance, deformation, damping, relaxation, yielding), and thermal (cooling, persistence) properties using proprietary scales. The aim of the work was to better understand which physical properties are related to haptic perception of surfaces for furniture applications.

## Methods

### Ethics

The experiment was conducted in accordance with the standards laid out in the Declaration of Helsinki (1964) and its later amendments. All participants gave written informed consent before taking part and the collected personal data were processed in accordance with the General Data Protection Regulation (EU) 2016/679 (GDPR). No sensitive personal information was collected, and no invasive methods were used. The study was assessed for ethical compliance through an internal process at RISE and was approved by senior management at the Division of Surface, Process and Formulation.

### Stimuli

**Fig. 1:**
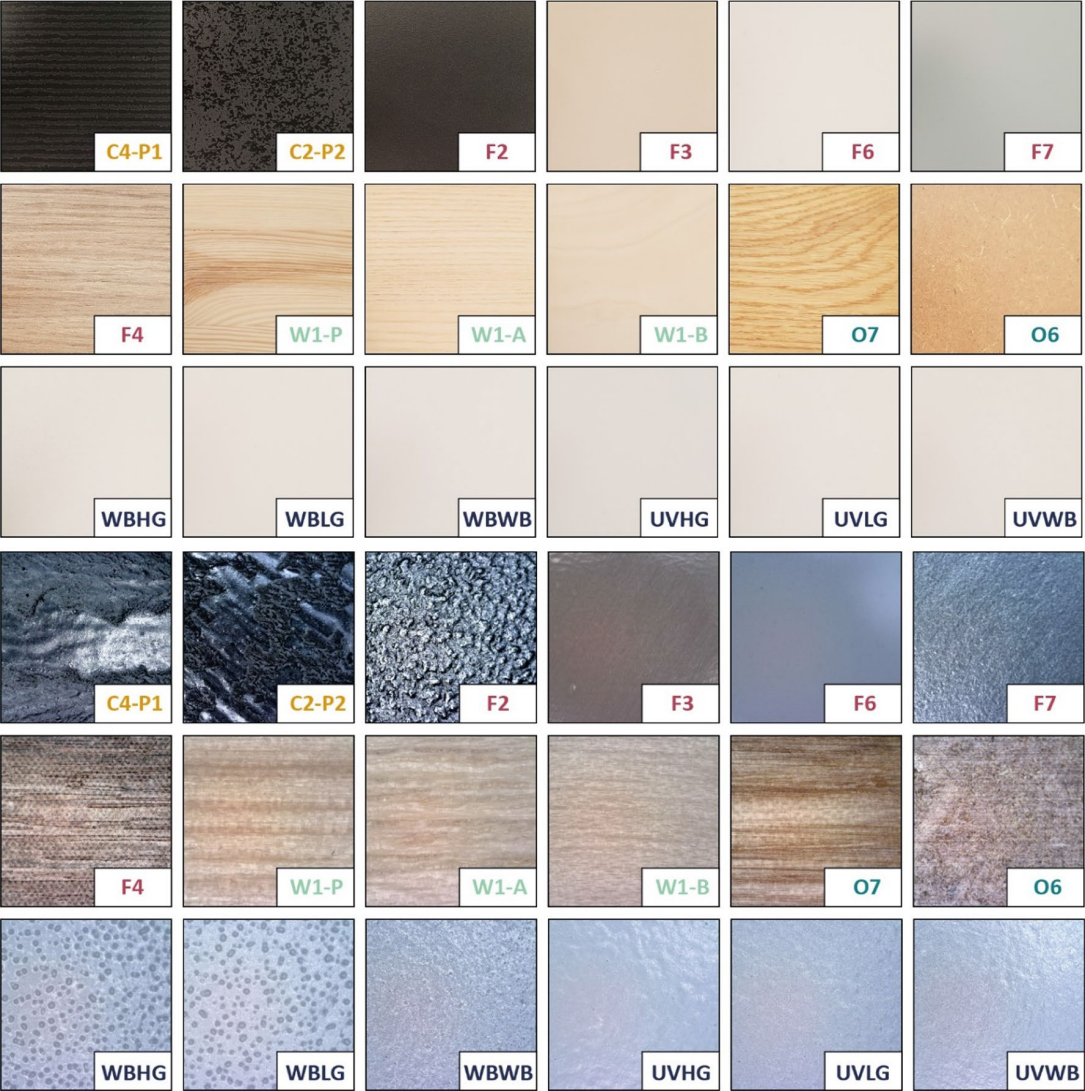
Top: optical images of 8 cm × 8 cm areas on selected examples of the stimuli included in the product landscape. Bottom: microscope images of the samples, 6 mm × 6 mm areas

Eighteen stimuli representative of a broad array of furniture finishes were selected and used in the study (see Fig. 1). The stimulus set included: two different embossed coating patterns (C4-P1 and C2-P2: radiation cured acrylates), five ‘foils’ (F2: embossed melamine formaldehyde resin on a paper foil; F3: radiation-cured acrylate; F6: PET foil with radiation-cured acrylate topcoat; F7: PP foil with radiation-cured acrylate topcoat; F4: melamine formaldehyde resin over a pressure laminate), three sanded (with decreasing sanding grain up to P320) but otherwise untreated natural woods (W1-A: ash, W1-B: birch, W1-P: pine), oiled natural oak, oiled MDF, and six variations of waterborne and radiation-cured primers with waterborne or standard waterborne radiation-cured topcoats with different textures. The samples varied in texture, surface/coating composition, color, and sheen and were intended to represent the breadth of surfaces consumers may encounter while selecting furnishing products for their own homes. The samples could be separated into several categories: embossed coatings (C4-P1, C2-P2), coatings with water-borne primers (WBHG, WBLG, WBWB), coatings with radiation-cured primers (UVHG, UVLG, UVWB), textured foils (F2, F4), glossy foils (F3, F6), natural woods (W1-A, W1-B, W1-P), and oiled woods (O6, O7).

### Physical characterization

Table 1 describes the relevant metrics from the physical characterization tests used and discussed in this work.

**Table 1:** Description and range across the samples tested of the relevant physical metrics in this study

	Description	Range across tested samples
Tactile friction coefficient (*μ*)	The ratio of the lateral force required to move the finger across the surface to the normal load applied	0.29–1.68
*R*_p_	The maximum peak height of the features over the sampling length	0.04–12.79 µm
*R*_dq_	The root mean square average slope of the surface profile	0.15–18.88°
*R*_sm_	The mean width of the profile elements	195.80–428.34 µm
*W*_ku_	The kurtosis of the waviness profile	1.08–1.85
SynTouch uRO	A time-varying signal indicative of vibration in a frequency band between 20 and 800 Hz	2.81–52.73
SynTouch tCO	A measure of how quickly heat is drawn from the biomimetic probe	14.73–24.16

#### Tactile friction

Tactile friction was measured using a ForceBoard™ (Industrial Dynamics AB, Sweden) a universal friction and force tester equipped with one horizontal and one tangential load cell (resolution 0.05 N, max capacity vertical: 10 kg, max capacity tangential: 5 kg), individually connected to the same plate of assembly. The mechanical loads are converted into voltage signals which are amplified and proportional to the applied load. The tangential force, i.e., friction force, and vertical force, i.e., applied load, were continuously recorded using DAQFactory software at a rate of 100 Hz as a finger was moved over the surface. The output data consisting of a text file of columns with the time and respective forces were further analyzed using MATLAB.

Friction measurements were performed by a single female operator and were repeated twice in the primary presentation direction (perpendicular to striated features when present) and once with the sample rotated 90°, giving three measurements per sample. This was to check for anisotropy in the friction character of the surfaces; however, none was found for any of the samples. Three different examples of each surface were tested in this manner. Each measurement consisted of ten reciprocating cycles at a normal load of approximately 1 N with the finger at an angle of approximately 30°. Dynamic friction coefficients were calculated as the ratio of friction force and applied load in each data point and the average friction coefficient of each stroke, measurement and surface type were calculated, not including the data points collected when changing direction of the finger.

#### Surface roughness

Using a Bruker Dektak XT with a stylus tip with a radius of 2 µm, area scans 2.0 × 2.0 mm^2^, comprised of 200 line scans at 10 µm intervals, were performed on one example each of the surfaces at a resolution of 0.25 µm/pt. Line scans 50 mm long were performed in three locations on three examples of each surface type at a resolution of 3 µm/pt, scanning in the same direction in which the samples were presented to participants. Roughness and waviness parameters were extracted using the Robust Gaussian Filter in Vision 64 Software with a 25 µm short wavelength cutoff and a 0.8 mm long wavelength cutoff, respectively, and a 10 mm sampling length. Cutoff wavelengths were selected based on stylus tip radius, visual inspection of the resultant roughness profiles as compared to the primary profile, and the success of a 0.8 mm long wavelength cutoff in other perceptual experiments (e.g., Fujiwara et al. 2005).

#### Biomimetic analysis of haptic dimensions

The stimuli were evaluated using a SynTouch Biotac Toccare™ with its default measurement profile, which returns numeric levels in arbitrary units for each of 15 dimensions of touch as described by SynTouch (for more information see Xu et al. (2013). The movement profile of the biomimetic finger includes shorter and longer sliding strokes at several loads, as well as several points of analysis at which the finger is stationary. Average values of five measurements per surface type were calculated for each dimension.

### Perception tests

20 female participants (*M*_age_ = 22 years, SD_age_ = 2 years) were recruited for the perception tests using an online recruitment system. All participants gave written informed consent before beginning the experiment. The samples were presented in pairs, and blindfolded participants were asked to judge the perceived similarity of each pair on a scale from 0 to 100%. Each participant performed 171 pairwise comparisons representing either the top or bottom half (ten participants each) of a matrix comprising of all possible stimulus pairs such that all pairs and presentation orders were evaluated within the scope of the experiment. In a second task, participants were asked to rank the suite of samples from least to most preferred in the context of use as a tabletop. They ranked the samples using touch alone, and then again using both vision and touch. The experimental session lasted 2.5–3.5 h, and participants were compensated with a gift certificate.

## Results

**Fig. 2:**
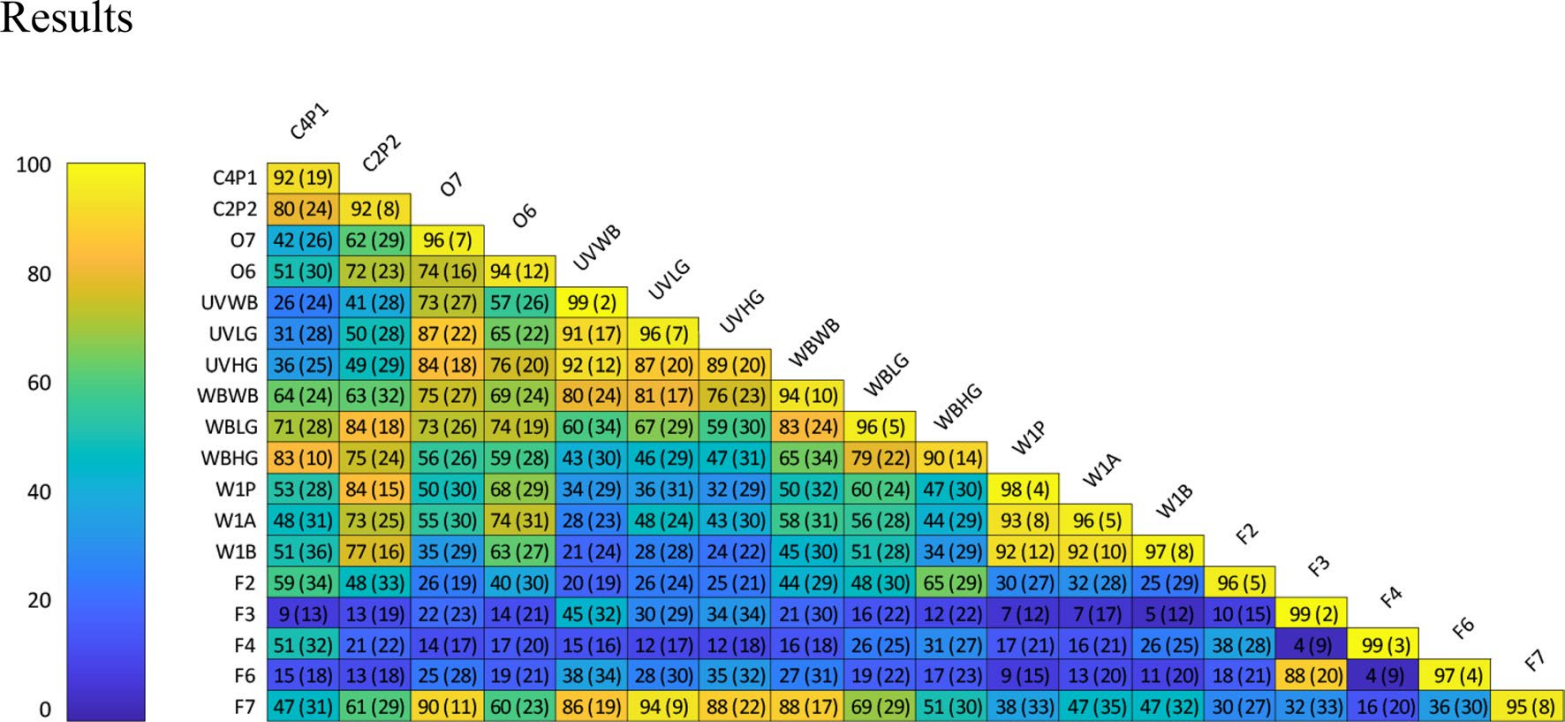
Mean perceived similarity (from 0 to 100%) for each pair of surfaces presented to participants. Values in parentheses are the standard deviations of the means. Tending towards yellow means that the two stimuli are perceived more similar and a blue colour indicates that they are perceived as more different. The values in the diagonal show average perceived similarity when the surfaces were compared against itself

**Fig. 3:**
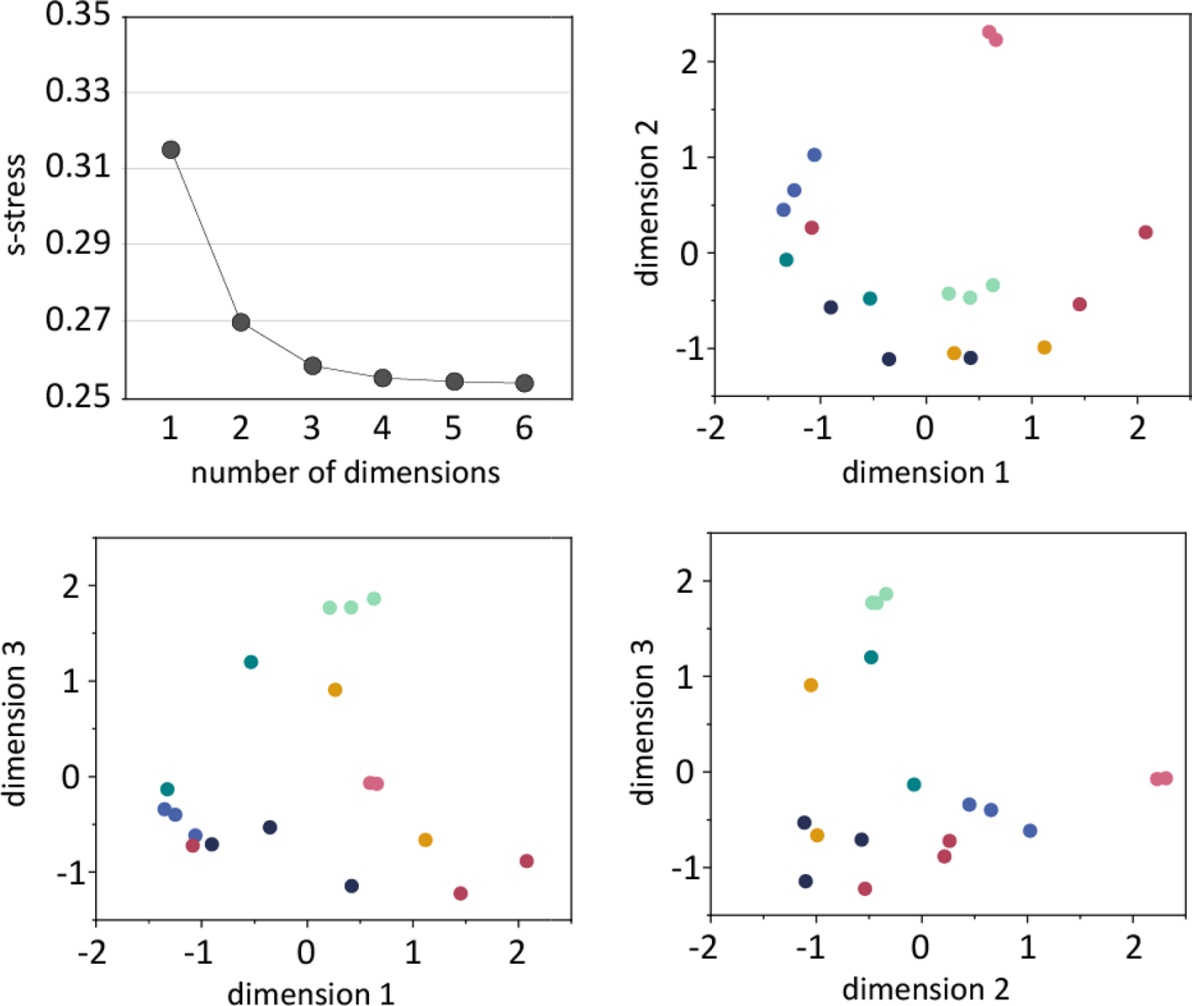
Upper left: scree plot showing the *s* stress values indicated by the INDSCAL analysis of the dissimilarity values. Remaining plots: The three haptic dimensions resulting from INDSCAL analysis of the dissimilarity matrices

Similarity values reported by the participants, see Fig. 2, were transformed to represent the dissimilarity of each pair by subtracting the values from 100. From the resulting dissimilarity matrices, inter-distances representative of the perceptual differences between the surfaces were mapped using Individual Differences Scaling (INDSCAL) in SPSS (version 25.0, IBM Corp). From the resulting scree plot (Fig. 3, upper left), it was determined that either a two-dimensional (*s* stress = 0.258) or three-dimensional (*s* stress = 0.270) solution could be used to describe the data. Both options were investigated. The three-dimensional solution (RSQ = 0.509) explained more of the variance than the two-dimensional (RSQ = 0.475) solution. Additionally, both dimensions in the 2D solution seemed to inadequately account for the position of the natural wood samples in the perceptual space, which were seen to be outliers in both dimensions. Thus, while the 3D solution had only a small improvement over the two-dimensional solution in terms of *s* stress, the elbow of the scree plot seems to be at dimension 3, the three-dimensional solution accounted for more variance in the data and better accounted for the wide variety of samples tested, as determined by the better fits in the analysis. Thus, we proceeded using the coordinates from the three-dimensional solution.

### Interpreting the haptic dimensions: results and discussion

The 3-dimensional solution is given in Fig. 3 and shows the distribution of the 18 furniture surfaces in the perceptual space. The closer the surfaces are situated in the map, the more similar they are perceived. Since it is difficult to visualize and interpret the dimensions when looking at this plot, in the following sections each perceptual dimension is plotted and analyzed separately to explain the physical cues relate to perceived similarity between the coated surfaces, and by extension, which physical properties are most prominent to modify if the tactile response should be controlled.

#### Dimension 1

**Fig. 4:**
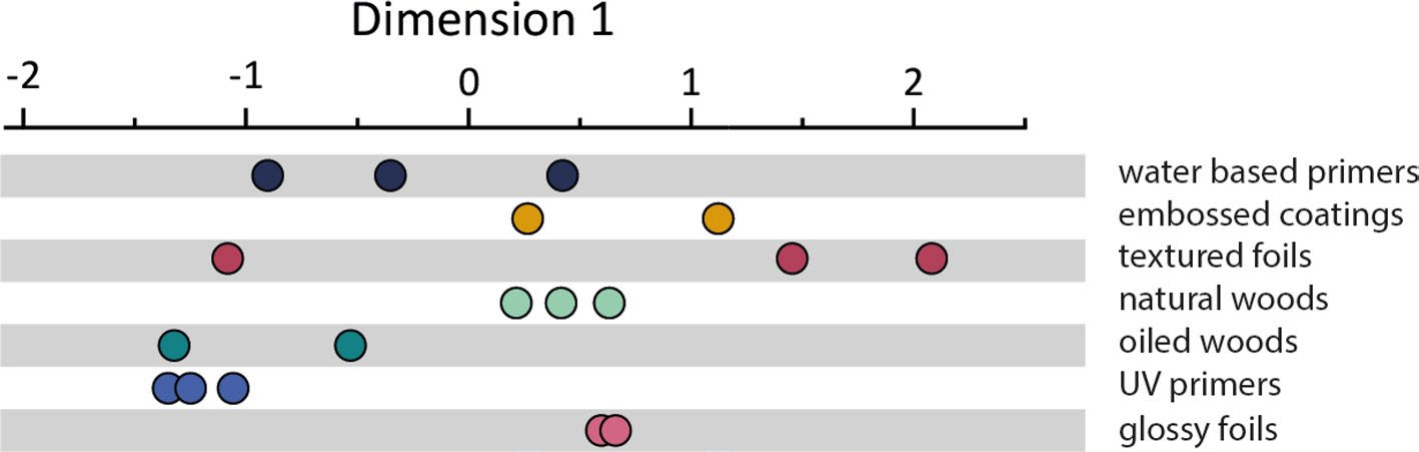
Spread of the stimuli in dimension 1, separated by category

The spread in dimension 1, separated by stimulus category, is shown in Fig. 4, where the stimuli are colored and separated along the *y* axis in arbitrary units to highlight the spread in dimension 1 and the proximity of samples with similar origin to one another. Samples in each category were not necessarily similar (e.g., the textured foils), but many were. Since the coefficient of friction is often found to be an important factor in discriminating surfaces (Gueorguiev et al. 2016; Skedung et al. 2013), we started by including this as a single factor in a linear regression on dimension 1. This returned a nonsignificant model (*R*^2^ = 0.073, *F*(1, 16) = 1.267, *p* = 0.28); however, the inclusion of *R*_p_ in the model as a second factor significantly improved the variance explained (Δ*R*^2^ = 0.720, Δ*F*(2, 15) = 28.770; *R*^2^ = 0.793, *p* < 0.001) and both *R*_p_ (*β* = 0.247, *p* < 0.001) and friction coefficient (*β* = 2.380, *p* < 0.001) became significant predictors. This suggests that the friction coefficient in this case may be acting as a suppressor of *R*_p_, possibly by controlling for variance in *R*_p_ that was not directly related to dimension 1. The further inclusion of *R*_dq_ (*β* = − 0.195, *p* = 0.005) again improved the model (Δ*R*^2^ = 0.066, Δ*F*(3, 14) = 6.540; *R*^2^ = 0.859, *p* = 0.023) and all three predictors were significantly associated with dimension 1, see Table 2.

**Table 2:** Unstandardized regression coefficients, *t* values and *p* values for dimension 1 revealed by INDSCAL

	*β*	*t*	*p*
Mean tactile friction	2.052	5.415	< 0.001
*R*_dq_ (°)	− 0.105	− 2.557	< 0.001
*R*_p_ (µm)	0.351	6.988	< 0.023

**Fig. 5:**
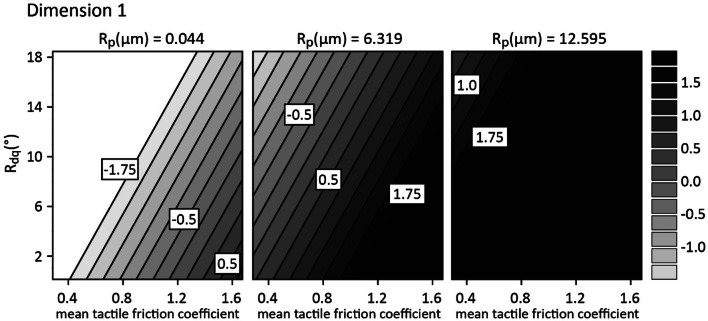
Contour plot illustrating the expected relative effects of tactile friction, *R*_dq_ and *R*_p_ on the spread of samples in dimension 1, i.e., the relevance of these parameters on the distinguishability of the surface pairs. The values on the contour lines show coordinates in dimension 1. The influence of the friction coefficient is seen at low *R*_p_, with some contribution from *R*_dq_ (left panel). When *R*_p_ increases to a medium level, both the friction coefficient and *R*_dq_ affect the distinguishability of the surface pairs (middle panel.). When *R*_p_ becomes high enough, neither *R*_dq_ nor the friction coefficient seem to influence the distinguishability of the surface pairs, as can be seen by the lack of contours here (right panel)

The first dimension of the 3D solution in this case was thus well described by a combination of the average tactile friction coefficient, the average maximum peak height (*R*_p_) and the root mean square average slope of the surface features (*R*_dq_). The expected effect of each of these three parameters on values in dimension 1 are illustrated in a contour plot in Fig. 5. At the lower boundary of *R*_p_ (left) the tactile friction coefficient is shown to affect the dimension 1 value for surfaces of relatively low slope. Even at low max peak height; however, a sufficient increase in *R*_dq_ is predicted to eliminate the effect of the friction coefficient. In the middle range of *R*_p_, increasing *R*_dq_ is expected to decrease the value in dimension 1 and increasing friction coefficient is expected to increase the value. At the upper bound of *R*_p_, the effect of friction coefficient and *R*_dq_ are almost eliminated suggesting that when the surfaces are sufficiently rough, other parameters become less relevant for haptic discrimination. The relative influence of the friction coefficient is thus governed by the roughness of the surfaces, and these parameters together explain a large proportion of the spread in this dimension.

Since roughness and friction are often presented as orthogonal dimensions for haptic touch, this result for dimension 1 requires some appreciation of the relationship between friction and roughness. For a very smooth surface, the friction coefficient will depend strongly on the chemical and mechanical character of the surface. An incremental increase in roughness has a tendency to decrease the friction coefficient up to a point, and the contribution of roughness to the friction force also depends on the size of the features, and on their shape, e.g., the average steepness or shallowness of the profile (characterised here by the parameter *R*_dq_). For instance, in samples of the same material, higher tactile friction could be expected from a surface with tall, sharp peaks as opposed to one with shallower, lower amplitude features—if the sharp peaks are large enough to instigate a ploughing mechanism during sliding, and if the material itself does not have a very adhesive interaction with skin. Complicating the effect, a human can detect both changes in roughness and in sliding resistance, though the sensation of ‘friction’ is likely to be overshadowed by the sensation of ‘rough’ (or at least a person may find it more intuitive to describe the sensation as one of roughness than of friction). In this study, participants have been observed to rate smooth, high friction surfaces that exhibit stick slip behavior during exploration as comparatively rough suggesting that, haptically speaking, one of these properties should not be investigated without some consideration of the other. This can be exemplified here by noting that both the glossy foils, which had the highest friction and lowest *R*_p_ of the samples, and the natural woods, which had the highest mean peak feature heights but the lowest tactile friction are found near the centre of dimension 1, see Fig. 4.

**Fig. 6:**
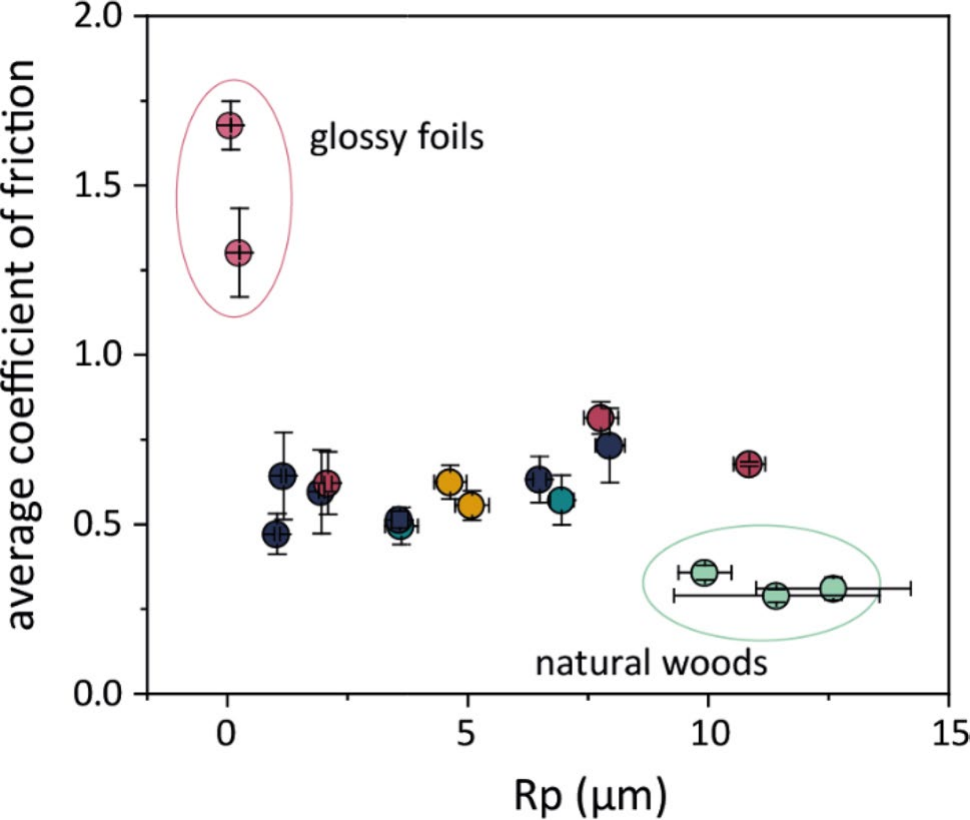
Average tactile friction coefficient of all the stimuli plotted against the maximum peak height (*R*_p_), demonstrating the complex relationship within this sample set. Error bars show ± one standard error of the mean

In the context of the stimuli included in this experiment, the relationship of the tactile friction coefficient with the roughness, in this case *R*_p_, can be observed to vary within some of the categories, but not directly with *R*_p_ (Fig. 6). In the case of the very smooth, glossy foils, the tactile friction coefficient is quite high—a combination of the effect of the low roughness and chemical character of the surface. In the midrange of *R*_p_, the stimuli demonstrated a slight, gradual increase in friction with increasing *R*_p_. The natural woods, however, despite being some of the roughest samples, had the lowest measured tactile friction coefficient.

When fitting haptic dimensions, it is often the case that certain roughness parameters and the tactile friction coefficient are used to describe separate dimensions. However, the fact remains that the friction coefficient depends not only on the material identity of the stimuli, but also on their roughness. It is, therefore, reasonable that measurements of friction and roughness might work together to describe one dimension of the haptic map. We tentatively suggest that dimension 1 here is also illustrative of the difficulty participants sometimes have separating friction and roughness, given their strong dependence on one another, and it is possible that this dimension is representative of the lateral deformations in the finger caused by the roughness–friction relationship.

#### Dimension 2

**Fig. 7:**
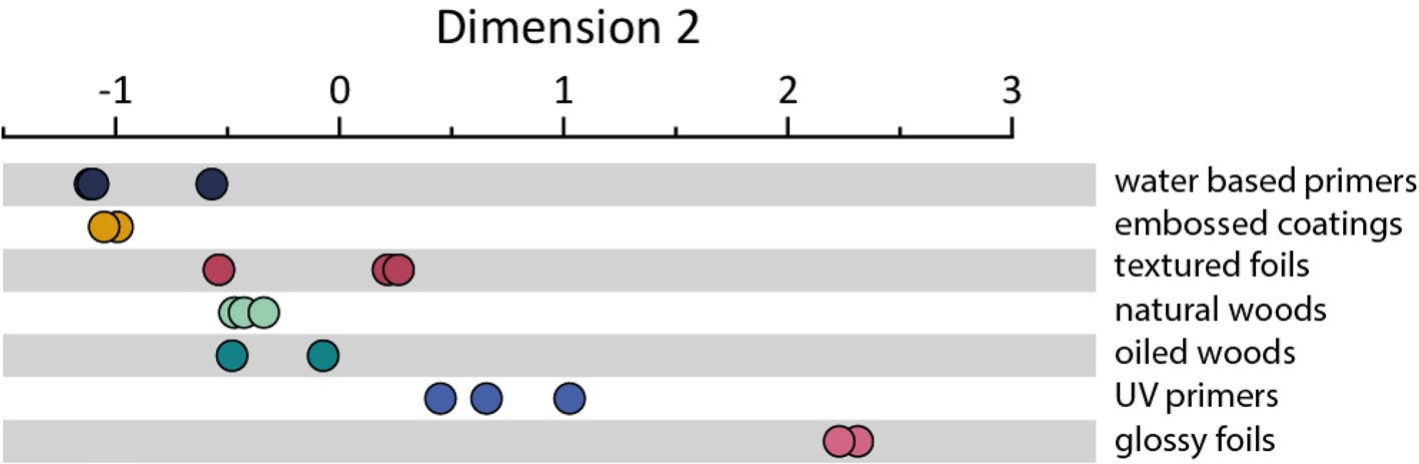
Spread of the stimuli in dimension 2, separated by category

As can be seen in Fig. 7, most immediately apparent when considering dimension 2 was that the stimuli appeared to group by category. Supporting this assertion, ANOVA indicated that the values in dimension 2 did indeed vary significantly by category, *F*(6, 11) = 36.44, *p* < 0.001. Further exploratory ANOVA suggested that the stimuli also varied significantly by category in several of the measured physical parameters, most notably in the SynTouch parameter uRO, *F*(6, 11) = 4.193, *p* = 0.019, the kurtosis of the waviness profile (*W*_ku_), *F*(6, 11) = 4.748, *p* = 0.013, and the average tactile friction coefficient *F*(6, 11) = 23.98, *p* < 0.001.

**Fig. 8:**
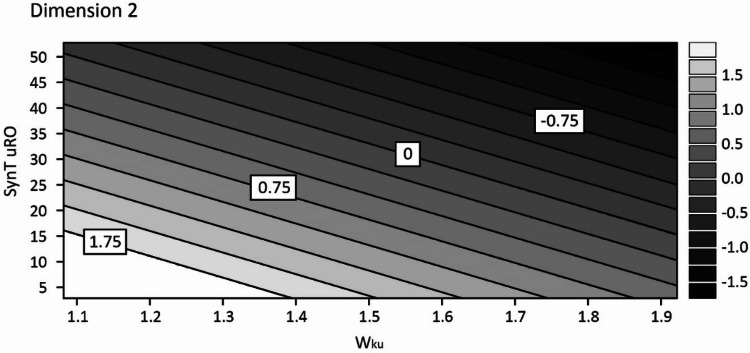
Contour plot illustrating the expected relative effects of SynTouch uRO and *W*_ku_ on the spread in dimension 2

Of the aforementioned variables, *W*_ku_ fit well as a single variable in a regression model on dimension 2 (*R*^2^ = 0.532, *p* < 0.001). Adding the friction coefficient as a second variable alongside *W*_ku_ in a model resulted in a significant increase in goodness of fit (Δ*R*^2^ = 0.205, Δ*F*(2, 15) = 11.69, *p* = 0.004; *R*^2^ = 0.737, *p* < 0.001) and both variables showed significant relationships with dimension 2 (*β* = 1.485, *p* = 0.004 and *β* = − 2.939, *p* = 0.002, respectively). However, the further addition of the uRO parameter to the model resulted in the effect of the friction coefficient becoming non-significant (*β* = 0.845, *p* = 0.057) suggesting that, in this case, uRO was able to explain the variance previously related to the friction coefficient. A Sobel test (on models excluding *W*_ku_) showed this to be the case (*z* = 2.67, *p* = 0.008). This contrasts with dimension 1, where the presence of friction enhanced the predictive power of the surface roughness. Thus, the final fit for dimension 2 included the kurtosis of the waviness profile and uRO, see Table 3 and Fig. 8.

**Table 3:** Unstandardized regression coefficients, *t* values and *p* values for dimension 2 revealed by INDSCAL

	*β*	*t*	*p*
*W*_ku_	− 2.137	− 2.860	0.012
SynTouch uRO	− 0.050	− 4.357	< 0.001

The spread in dimension 2 is best described by the vibration based uRO parameter and a shape parameter from the waviness profile, *W*_ku_

The biomimetic sensor in the SynTouch BioTac Toccare™ produces a time-varying signal indicative of vibration, and for uRO a measure of vibration intensity in a frequency band between 20 and 800 Hz is determined by the instruments internal analytical function. Interestingly, uRO was a better fit for this model than any of the direct roughness measurements by the profilometer. The SynTouch assesses roughness by analyzing induced vibrations in the synthetic fingertip rather than by measuring the exact variations in height as the stylus does, we tentatively propose that dimension 2 may be related to the vibrational aspects of tactile exploration of the stimuli due to both the microscale roughness (small amplitude, high frequency events) and the larger scale waviness, which may introduce a longer wavelength vibratory component or simply contribute a distinct perception of ‘shape’ in addition to the smaller scale roughness. This is further supported by the finding that the friction coefficient was not found to significantly contribute to the description of dimension 2 in the presence of roughness parameters (*W*_ku_ was not related to the tactile friction coefficient after the bimodality of the relationship due to F3 and F6 was accounted for). Figure 8 shows the relationship between *W*_ku_ and uRO and their effect on sample placement in dimension 2. Each parameter affected dimension 2 at all values of the other, with decreases to both increasing the position in dimension 2.

#### Dimension 3

**Fig. 9:**
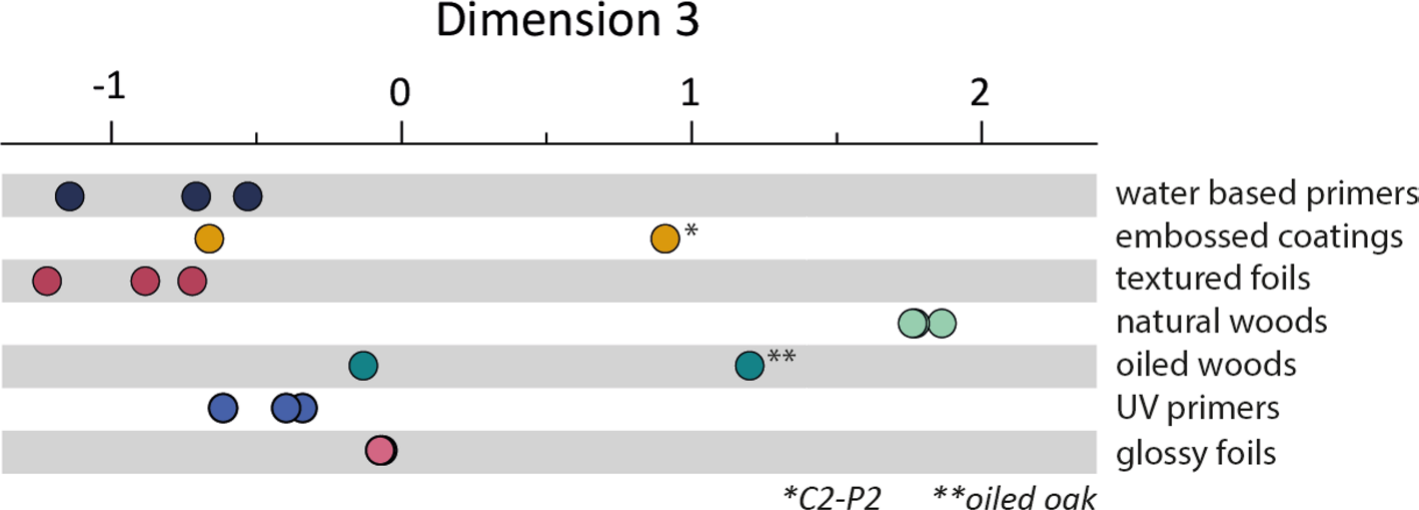
Spread in dimension 3 by surface category

As can be seen in Fig. 9, in dimension 3, the natural woods (light green) and one of the heavily embossed coatings (C2-P2) stand out as relative outliers, placing very high in dimension 3. This suggests that this dimension might be related to physical features peculiar to natural woods. Indeed, significant correlations between dimension 3 and the SynTouch parameter tCO (*r* = − 0.745, *p* < 0.001), *R*_dq_ (*r* = 0.636, *p* = 0.005) and the mean width of the profile elements, *R*_sm_ (*r* = − 0.529, *p* = 0.024) were found. Including all three of these parameters in multiple regression resulted in a significant model (*R*^2^ = 0.821, *F*(3,14) = 21.45, *p* < 0.001) (Table 4).

**Table 4:** Unstandardized regression coefficients, *t* values and *p* values for dimension 3 revealed by INDSCAL

	*Β*	*t*	*p*
SynTouch tCO	− 0.160	− 2.864	0.012
*R*_dq_ (°)	0.090	3.612	0.003
*R*_sm_	− 0.007	− 3.534	0.003

The natural woods were relative outliers in tCO (a measure of how quickly heat is drawn from the biomimetic probe), *R*_sm_ and *R*_dq_, suggesting that this model does not explain the differences between all the stimuli in this dimension, but rather the differences between the natural wood group and the other stimulus categories. It also suggests that the textured coating C2-P2 at least in some respects closely approximates the feeling of natural wood. This representation of the perceptual uniqueness of natural wood was a key motivation for using the 3-dimensional solution instead of the 2-dimensional solution, where the natural woods were difficult to fit satisfactorily. The natural periodic structure of wood grain is a strong contributor to the distinct values of *R*_sm_, which are related to the cell structure and sanding of the wood species (Kúdela et al. 2018), wood is often described as being of distinct thermal character, or as ‘warm’, (Obata et al. 2005; Wastiels et al. 2012) and *R*_dq_ acts as a descriptor of the shape of the profile elements_._ These three components together create a compelling approximation of some of the factors that may describe the perceptual uniqueness of natural wood surfaces.

**Fig. 10:**
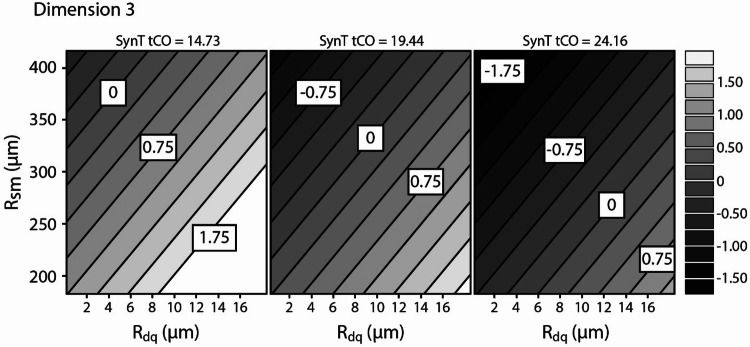
Contour plot illustrating the relative effects of *R*_sm_ and *R*_dq_ on the spread in dimension 3, at three levels of tCO. Decreases in *R*_sm_ and *R*_dq_ are associated with a feeling closer to natural wood (more positive in dimension 3). Also, when heat is drawn more slowly from the finger (left panel) surfaces are expected to more closely resemble natural wood. The plot shows that decreases in tCo, *R*_dq_ and *R*_sm_ are expected to all be related to surface more closely resembling natural wood

Keeping this bimodality in dimension 3 in mind, we constructed a contour plot for dimension 3 based on the three variables in the final model, see Fig. 10. This plot illustrates that decreases in the mean profile element width (moving toward more narrow elements) and tCO (slower to draw heat from the finger), and increases in *R*_dq_, are expected to lead to changes in dimension 3 towards the values of the natural woods. This suggests that if seeking to mimic the tactile quality of natural wood, manufacturers should consider the shape and spacing of the features of their created surfaces, rather than the average roughness or appearance alone, and that the thermal properties of the surfaces also play a role in differentiating mimics from the real thing.

### Preference rankings: results and discussion

In addition to the similarity scaling task, participants were asked to rank the stimuli from most to least preferred in the context of being a tabletop; first by touch only, and then by combined touch and vision. Stimulus rankings were subtracted from 19 to yield ‘points’, giving 18 for the sample ranked most preferred and 1 for the sample ranked least preferred. Scores were averaged for each stimulus.

**Fig. 11:**
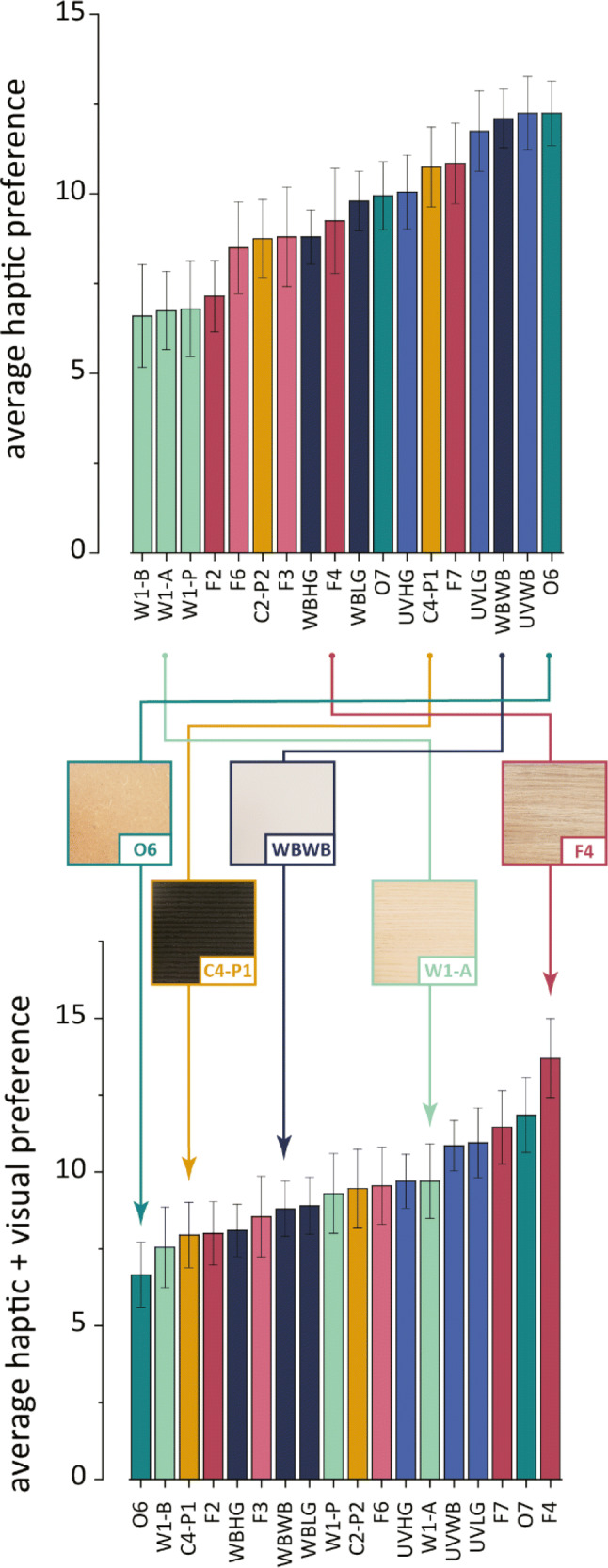
Bar plots showing the average preferability points that each surface received in a ranking task (high preference receives more points) by **a** haptic exploration only, and **b** combined visual and haptic exploration. Error bars show ± one standard error of the mean. Surfaces whose preferability changed dramatically are indicated graphically

Preferability scores in the touch-only section of the task correlated with the values in the first and third haptic dimensions from the INDSCAL analysis (*r* = − 0.659, *p* = 0.003; *r* = 0.647, *p* = 0.004). Recalling that dimensions 1 and 3 were, in different ways, related to the average tactile friction coefficient, *R*_dq_, *R*_p_, tCO and *R*_sm_, a significant model was reached from a combination of these factors (*R*^2^ = 0.936, *F*(3, 14) = 68.09, *p* < 0.001) namely the tactile friction coefficient, *R*_p_, and tCO, see Table 5. This model suggests that all three types of tactile information discussed (roughness, friction, thermal properties) were relevant, and the most preferred surfaces were in general smoother, with lower friction, and higher thermal cooling coefficients as measured by the SynTouch, see Fig. 11. This analysis was not conducted for rankings by touch plus vision, because we did not quantify physical parameters relevant for visual inspection of the surfaces, e.g., luminosity, gloss etc., and so we concluded that an adequate model would not be found here.

**Table 5:** Regression coefficients, *t* values and *p* values for the preference rankings by touch alone

	*β*	*t*	*P*
SynTouch tCO	0.479	8.539	< 0.001
*R*_p_ (µm)	− 0.357	− 9.576	< 0.001
Mean tactile friction	− 3.870	− 8.403	< 0.001

The rankings of the surfaces from the touch only task did not correlate with rankings, where vision was included (*r* = 0.155, *p* = 0.540) suggesting some differences between haptic and visual preferences. Indeed, preference for several of the samples saw comparatively large changes when vision was included. The natural woods were generally more preferred when visible, and the imitation wood foil, F4, moved from the middle of the pack to the most favored surface, on average. The embossed, striated coating C4-P1 lost relative appeal once visible, and the water-based primer and radiation cured primer coatings rearranged to be much closer to the other members of their respective groups. The oiled MDF was most negatively affected by introducing vision, moving from most preferred by touch, to least preferred by vision and touch combined. The difference between the two tasks demonstrates that preferences change depending on the sensory information available, i.e., whether from touch alone or a combination of vision and touch. It should be noted here that the colors and patterning of the surfaces were different which may have influenced the preferences by vision.

### General discussion

Our sense of touch is hierarchical and complex, and humans are remarkably sensitive to differences between surfaces. Roughness and friction (or stickiness/slipperiness) are commonly cited as major dimensions of haptic perception (Okamoto et al. 2013). In this work, using a broad selection of surfaces, we have shown that the two are closely related, and the relative salience or relevance of these factors on tactile perception seems to depend on the availability of other cues, e.g., feature shape or distribution, as well as the material properties of the surfaces in question.

For the surfaces included here, people’s perceived similarity of the surfaces could be adequately explained by three haptic dimensions. These dimensions, however, did not pertain to variations in only one physical feature, and instead were found to be, in themselves, complex. Dimension 1 could be broadly described as a friction-roughness dimension, where the influence of the peak feature heights was enhanced by frictional cues and shows one way in which roughness and friction can be perceptually confounded by participants. Dimension 2 initially appeared to be related to friction but instead seems to represent the vibrational character as well as the waviness profile of the surfaces. Finally, dimension 3 appeared to capture the ways in which natural wood is differentiable from synthetic materials and was related to variance in how heat is drawn from the finger, the profile element width as well as the distribution of the surface features.

The result that people tended to prefer the low friction surfaces when ranking preference by touch alone supports previous findings, where tactile preference and tactile friction have been negatively associated (Klöcker et al. 2013; Skedung et al. 2018). We also found that preferences differed depending on whether judgements were made by touch alone or by touch and vision. Interestingly, Vardar et al. (2019) found strong correlations between similarity ratings of surfaces by vision and by touch, suggesting that texture perception may be similar across the two modalities. The results here, however, suggest that although differentiation may be similar across the two modalities, differences might be found when preferences are measured instead.

In their study, Vardar et al. (2019) also used perceived similarity and multidimensional scaling to investigate the physical parameters relevant for haptic and visual differentiation of a range of surfaces. Their three-dimensional solution similarly highlighted the importance of friction, roughness and vibrational information, as well as hardness, for surface differentiation by haptic touch, but in contrast to this work, these were more independent. It is likely that differences between their stimuli, which varied widely, and those tested here account for at least some of this difference. In Fig. 5, both the glossy foils (high tactile friction and low *R*_p_) and the natural woods (high *R*_p_ and low tactile friction) are found near the center of dimension 1. This indicates that the samples used here covered a perceptual grey area, where friction and roughness provide tactile sensations that, to the perceiver, were not meaningfully distinguishable. A similar effect was reported by Arvidsson et al. (2017), see also Skedung et al. (2013), where flat, high friction surfaces were scaled as feeling rough by participants. Such a crossover between friction and roughness did not seem to be present in the results of Vardar et al. (2019). The results found here thus both support previous findings that friction, roughness and (although indirectly measured here) vibrational information are important cues for differentiating surfaces by touch, and simultaneously re-iterate the finding that friction and roughness do not necessarily always describe orthogonal, independent dimensions of touch. As has been previously suggested by Bergmann Tiest and Kappers (2006), roughness perception may be associated with both friction and roughness.

As mentioned above, the role of vibrations in the present work can only be discussed somewhat speculatively, since the physical measurement was indirect. Nonetheless, for dimension 2, the possible influence of induced vibrations within the fingertip is noteworthy, because vibrations are an additional component of perception that are strongly affected by surface texture, or microslips, at the interface due to adhesion. Vibration information is particularly relevant for detection of microroughness (spatial period <  ~ 200 µm; Bensmaïa and Hollins 2003) and in fact humans are unable to reliably distinguish between very fine stimuli in the absence of vibrations induced by movement (Hollins et al. 2000; Hollins and Risner 2000). The exploration of this idea here was relatively simplistic and based on the fact that the final model for dimension 2 included uRO obtained from the SynTouch BioTac Toccare©, which is based on induced vibrations and is related in part to surface roughness (Ding et al. 2017) as compared to the roughness parameters as measured by the profilometer. In other work, the role of vibrations has been studied more in-depth, investigating the role of the period of the roughness as compared to finger ridge width (Fagiani et al. 2012) and it has been posited that the discriminability of different textures correlates better with the vibrations induced during sliding, rather than to the physical roughness of the surfaces (Cesini et al. 2018).

Additionally, dimension 2 seemed to be related to the waviness parameter *W*_ku_. It is not often that waviness parameters are discussed in relation to haptic perception, with researchers tending to focus on roughness height parameters such as *R*_p_ or *R*_a_ instead, and so some discussion of what this parameter means is necessary. The kurtosis of a profile (*λ*_c_ = 0.8 mm) is usually considered a measure of how peaked a surface is, with particular sensitivity to isolated peaks. A profile with kurtosis greater than 3 is considered ‘peaked’. More specifically, the kurtosis is a description of the weight of the tails of a distribution: kurtosis values below 3 describe distributions with shorter, thinner tails than a normal distribution and a broader center peak. All of the stimuli in this experiment had values of *W*_ku_ between 1 and 2, with 1 being the minimum possible value, meaning that the heights of the waviness profiles had similar amounts of values at each height increment, rather than a tendency toward being highly peaked. Regardless, the relative ‘peakedness’ of the profiles may be said to increase as the values approach 3, and it is evident that the second dimension of perceived similarity between these surfaces is strongly related to this descriptor the shape of the larger wavelength features of the stimuli. However, the waviness profiles used in this analysis included all wavelengths above 0.8 mm, which means that any form effects due, for example, to bowing of the surfaces are also included in the calculations. Further investigation of larger wavelength features, possibly from additional filtering passes, would be beneficial to eliminate the effects of form in future work.

It is noteworthy that dimensions 1 and 2 appear to reflect the coarse and fine aspects of the duplex theory of texture perception (Katz 1925), which suggests that our sense of touch depends on both a spatial sense and a vibratory sense. Surface feature size and the friction coefficient, found to be relevant for dimension 1, have a strong effect on the deformation felt in the fingertips during exploration, and specially adapted Merkel nerve endings and Ruffini corpuscles in the fingertips respond to the resulting skin stretch and pressure (Johnson 2001). The vibratory signals induced through movement across the surfaces, represented by uRO in dimension 2, are detected at low frequency by Meissner Corpuscles (10–50 Hz) and Pacinian corpuscles (30–150 Hz; Johnson 2001).

Our third haptic dimension appeared to sequester natural wood surfaces and was well described by differences in the rate at which heat may be drawn from the finger and several feature shape parameters. It is, unfortunately, unclear whether participants directly perceived these thermal differences, which may also have been partly related to roughness, or whether these parameters were simply those in which natural wood surfaces were the most unique. Regardless, this third dimension sheds some light on both the context sensitivity of dimensional analysis, as well as on the unique haptic character of uncoated wood surfaces, which is difficult to replicate synthetically.

Deviations from natural wood through coating may be cumulative, and impact on not only the perceived preference of surfaces but also on key physiological responses to touching those surfaces. In one study, Ikei et al. (2017) compared various physiological responses to touching uncoated, oil-finished, vitreous-finished, urethane-finished, and mirror-finished white oak wood (written in increasing order of processing and treatment, and thus physical deviation from uncoated wood). These authors showed that touching uncoated wood was associated with an increase in parasympathetic nervous system activity (i.e., a relaxation effect) relative to touching mirror-finished, vitreous-finished and urethane-finished wood, calmed left prefrontal activity relative to mirror- and urethane finished wood, and decreased heart-rate relative to mirror-finished wood. The fewest differences in physiological response were found between the uncoated and oil-finished woods, which were also the most similar in physical character, including roughness and thermal conductivity. These results indicate that increasing the amount of processing and thus distance from a natural-wood feeling may be associated with increasing differences in physiological response to these surfaces.

Retaining the perceived naturalness of wood surfaces intended for interior use while also ensuring durability and easy cleanability is a challenge within the furniture industry. This is complicated further by the desire to simultaneously retain a feeling of naturalness despite processes to improve cleanability and durability while additionally ensuring the product feels pleasant to consumers. Our participants tended to prefer smoother, low friction surfaces which draw heat from the finger, but it is not yet known whether these factors also contribute to a feeling of naturalness. Since the natural wood samples were the least preferred by touch alone and benefitted the most in terms of preference ratings when the participants could also see the stimuli, the relationship between naturalness and preference may not be simple. Overvliet and Soto-Faraco (2011) used different psychophysical methods to show that an underlying construct of naturalness can be accessed, and that both vision and touch contribute to a visuo-tactile perception of naturalness. It would be fruitful to more closely examine the relationship between perceived naturalness and preference/pleasantness in future work as well as analyzing these with respect to physical parameters of both natural and synthetic materials.

The aforementioned difficulty in confidently interpreting whether factors such as the cooling properties of the surfaces were indeed detected by participants highlights one of the limitations of the present work, namely the exclusion of descriptive or sensory evaluations, for example using a semantic differential task, of the surfaces by participants. In future work, a task will be included to further improve the interpretation of the underlying haptic dimensions relevant for discrimination, as well as better understand how participants interpret terms such as rough, smooth, and slippery. As previously discussed, this issue can become complex quite quickly when asking questions such as whether a slippery surface is necessary smooth, or whether softness truly is the opposite of hardness, and when the same words may be interpreted differently depending on the stimuli presented.

In conclusion, the present work demonstrates that variations in friction and roughness influence tactile perception of surfaces in complex ways, both due to their relationship to each other as well as the inherent complexity of human perception. When a surface is smooth, resistance to sliding may still be perceived. As roughness increases, the cause of the resistance to sliding shifts from purely chemical (on a perfectly smooth surface) to a mixture of material identity and feature size, shape, and spacing which affect the mechanical interactions between the finger and the surface (on rougher surfaces). We suggest that expanding characterization of roughness in a standard laboratory setting to include parameters other than the most commonly used height parameters (*R*_a_) may allow for better quality control and predictive steering of haptic qualities of materials.
